# Treatment satisfaction among patients in opioid agonist treatment in Norway: A multicenter cohort study

**DOI:** 10.1177/14550725251351711

**Published:** 2025-07-09

**Authors:** Linda Nesse, Thomas Clausen

**Affiliations:** Norwegian Centre for Addiction Research, Institute of Clinical Medicine, 60504 Faculty of Medicine, University of Oslo, Oslo, Norway; Norwegian Centre for Addiction Research, Institute of Clinical Medicine, 60504 Faculty of Medicine, University of Oslo, Oslo, Norway

**Keywords:** coordinated care, opioid dependence, opioid maintenance treatment, patient satisfaction, quality of care, treatment experiences, service satisfaction

## Abstract

**Background:** Treatment satisfaction is an important indicator of patients’ perceptions of opioid agonist treatment (OAT) for opioid dependence. In addition, treatment satisfaction may have implications for treatment outcomes. The present study aimed to explore treatment satisfaction among OAT patients one year after enrollment, as well as the role of different psychosocial and treatment characteristics in treatment satisfaction. **Methods:** The study was based on data from the NorComt project (2012–2016), which used a multicenter cohort design with structured questionnaire-based interviews. The sample consisted of 175 OAT patients in Norway who rated their treatment satisfaction (high, mixed, low) one year after enrollment in a new treatment episode in OAT. Treatment satisfaction (high versus mixed or low) was used as a dependent variable in logistic regression models, with demographic characteristics, well-being, substance use, and treatment characteristics as independent variables. **Results:** Overall, 54.9% of the participants reported high satisfaction with OAT one year after enrollment, while 25.7% reported mixed satisfaction and 19.4% low satisfaction. Of the 175 participants who reported on treatment satisfaction, 161 remained in treatment at follow-up. Reporting an active occupational status and an individual treatment plan in OAT was associated with increased odds for reporting treatment satisfaction, while reporting ongoing substance use was associated with decreased odds for reporting treatment satisfaction. **Conclusions:** Psychosocial, situational factors, as well as coordinated, integrated support, may play a role in OAT patients’ treatment satisfaction. To identify facilitators and barriers in OAT, it is vital to address relevant needs and goals for treatment from the perspectives of patients.

## Introduction

Treatment satisfaction is a multifaceted construct that refers to patients’ self-reported levels of satisfaction and perceptions of treatment (De Maeyer et al., 2009; Miglietta et al., 2018). As such, treatment satisfaction is an important indicator of the subjective experience of treatment modalities such as opioid agonist treatment (OAT) for opioid dependence (Trujols et al., 2012). OAT encompasses medical treatment, as well as psychosocial support and interventions, and aims to improve quality of life and living conditions (e.g., relationships, economy, housing, and activities) and reduce health harms and overdose risk among individuals with opioid dependence.

Given an increased emphasis on patient perspectives and user involvement, it is vital to assess treatment satisfaction, and to utilize the knowledge gained to inform and improve treatment practices (De Maeyer et al., 2009). In services and research, treatment satisfaction is often assessed through patient-reported experience measures, utilized to systematically collect feedback from patients regarding their treatment experiences (Migchels et al., 2023).

In the case of opioid dependence, assessment of treatment satisfaction becomes especially relevant because negative views of OAT have been identified as a substantial barrier to treatment, potentially hindering enrollment as well as retention (Hall et al., 2021). According to a recent review of treatment satisfaction in mental health services, patients who report higher satisfaction tend to more actively engage and remain in treatment (Windle et al., 2020). This also appears to be applicable to OAT (Marchand et al., 2011).

Treatment satisfaction in OAT and related modalities, such as heroin-assisted treatment, may vary across treatment dimensions, and satisfaction with individual dimensions may influence overall satisfaction (Ellefsen et al., 2023; Marchand et al., 2022). Patients in OAT have, for example, highlighted consistent service provision, accessible psychosocial support, person-centered support, involvement in decision-making, and satisfaction with OAT medications as appreciated aspects of treatment (Marchand et al., 2022; Trujols et al., 2012; Vanderplasschen et al., 2015). Conversely, inconsistent regulations, negative interactions with treatment providers, treatment burden, inadequate dosing, and experienced side effects of OAT medications have been cited among the aspects of OAT that may reduce satisfaction (Marchand et al., 2015, 2022; Muller et al., 2018; Trujols et al., 2017). Lower levels of satisfaction may partially reflect unmet treatment expectations or needs, such as the need for mental health treatment or involvement in meaningful activities (Apantaku-Olajide et al., 2012; Vanderplasschen et al., 2015). In addition, some studies have found variations in treatment satisfaction depending on characteristics such as age and gender (Marchand et al., 2015), appraisal tendencies (Hansson et al., 2007), and subjective quality of life and well-being (Marchand et al., 2022; Miglietta et al., 2018). Treatment satisfaction may potentially evolve during treatment, provided that important needs are sufficiently and appropriately addressed (Müller et al., 2020). Treatment needs may be tackled through agreement between a patient and their treatment provider regarding personally relevant treatment goals and the provision of systematic, coordinated support (Friedrichs et al., 2016). For patients in OAT, indicators of successful treatment and recovery may include social stability, reduced substance use, enhanced well-being, and an improved sense of self (Hooker et al., 2022; Reed et al., 2023).

Given that treatment satisfaction is an important indicator of successful treatment in OAT, the present study aimed to explore treatment satisfaction among a cohort of patients in OAT in Norway one year after enrollment in a new treatment episode, and to explore the role of a set of psychosocial and treatment characteristics in treatment satisfaction.

## Methods

### Design

This multicenter cohort study is part of the Norwegian Cohort of Patients in Opioid Maintenance Treatment and Other Drug Treatment (NorComt) research project (e.g., Medved et al., 2020; Muller et al., 2016). Data were collected from 14 OAT units in Norway between 2012 and 2016, at enrollment in a new treatment episode (baseline), and approximately one year after enrollment (follow-up). Because treatment satisfaction was only assessed at follow-up, this study is primarily based on follow-up data.

### Study setting

The Norwegian OAT population consists of approximately 8,500 patients (30% women). It is estimated that approximately 80% of persons with opioid dependence in Norway are enrolled in OAT (European Monitoring Centre for Drugs and Addiction, 2023). OAT in Norway is free of charge and publicly funded. OAT is delivered in outpatient units within specialized substance use treatment, in collaboration with municipal services and general practitioners, to ensure integrated, interdisciplinary treatment. The medical treatment component in OAT includes a range of medications, individual dose adjustments, medical follow-ups, and safety measures. In addition to the medical treatment, psychosocial support is available. Psychosocial support and interventions in OAT may include tailored individual treatment plans and patient care teams, or other relevant approaches involving all relevant stakeholders to secure individualized and coordinated treatment, housing support, activity and employment support, financial support, as well as optional mental health treatment (Norwegian Directorate of Health, 2017, 2018). In terms of treatment goals, approximately two-thirds of patients have reported aim for rehabilitation (i.e., cessation of substance use), while the remaining third aims for harm reduction (i.e., decreased substance use and associated harms) (Nesse et al., 2024).

Each year, OAT patients in Norway are asked to participate in a national annual survey mapping their current treatment status. In the most recent annual survey, conducted by treatment providers across OAT units, responses regarding treatment satisfaction indicated that 59% were satisfied with their treatment, 16% had mixed treatment satisfaction and 4% were dissatisfied, with missing data for 21% of patients (Nesse et al., 2024). Missing data on treatment satisfaction are interpreted to likely reflect a high proportion with low satisfaction. The distribution of satisfaction scores has remained relatively stable in the overall patient population over the past decade, although it is not known how treatment satisfaction develops within individual patients over time, nor across treatment dimensions.

### Procedures and participants

Patients who were enrolled in OAT within the past four weeks at baseline were eligible for participation in the research project if they had a diagnosis of opioid dependence in accordance with the ICD-10 criteria. Recruitment was carried out by treatment providers, who also screened patients for study eligibility. At baseline, 438 OAT patients were eligible for participation, of whom 283 participated (65% response rate). Of the 283, 179 participated at follow-up (63% response rate). The final sample consists of the 175 of the participants who rated their overall treatment satisfaction in OAT at follow-up. Structured interviews were carried out by OAT providers at baseline (at the OAT services, approximately four weeks after enrollment in treatment) and researchers at follow-up (at various locations, approximately one year after enrollment). Participants received a gift card at follow-up to compensate for their contribution (approximately €30).

### Measures

The interviews were based on self-report questionnaires that included items on treatment satisfaction, demographic characteristics, substance use, well-being and treatment characteristics. *Treatment satisfaction* was operationalized as satisfaction with the treatment at large, formulated as “Altogether, how satisfied are you with the treatment?” (high satisfaction, mixed, or low satisfaction) (Muller et al., 2016).

*Demographic characteristics* included gender, age at baseline, occupational status (employed and/or studying) and housing situation (housing stability within the past four weeks) one year into treatment.

*Substance use* was assessed with items on ongoing substance use (yes/no), number of substances used, intravenous substance use (yes/no) and involvement in a substance-using social network (yes/no), all within the past six months.

*Well-being* was assessed with three items from a quality-of-life measure using a five-point Likert scale (Muller et al., 2016). These items were current relationship with self, relationship with friends, quality of contact with family (0 = very poor, 4 = very good). In addition, well-being was assessed with a single item on experienced severe anxiety symptoms within the past four weeks (yes/no) and a single item on experienced severe depressive symptoms within the past four weeks (yes/no).

*Treatment characteristics* was operationalized using medical and psychosocial variables. The medical variables included type of medication (methadone, mono buprenorphine, or buprenorphine combined with naloxone), supervised dispensing (0–4 or 5–7 times per week), weekly drug screenings (yes/no or missing), having had an OAT medication check-up within the past year (yes/no or missing) and having had a medical check-up with a general practitioner within the past year (yes/no or missing). As for psychosocial treatment variables, these included having received mental health treatment within the past year (yes/no or missing), the use of an individual treatment plan within the past year (yes/no or missing) and having had meetings with a coordinated patient care team within the past year (yes/no). In addition, items on treatment interruptions during the past year (no interruptions/at least one interruption) and current treatment status (currently in OAT/currently not in OAT) were included.

### Statistical analysis

Descriptive statistics, including frequencies, percentages and/or mean scores, and corresponding standard deviations were reported for the patient and treatment characteristics. Crosstabulations were used to compare participants across satisfaction groups. A chi-squared test (or Fisher's exact test in the case of small group sizes and Student’s *t*-test in the case of continuous variables) and corresponding *p*-values were calculated. Treatment satisfaction (low/mixed or high) was used as a dependent variable in the logistic regression analyses, and low and mixed satisfaction were combined as a result of small sample size. The logistic regression models included age at baseline (continuous), gender, occupational status (currently unemployed/currently employed), housing situation (unstable/stable), ongoing substance use (not using/using), involvement in a substance-using social network (no/yes), quality of contact with family (good/poor or mixed), individual treatment plan (yes/no), treatment interruptions during the past year (yes/no) and current treatment status (not in treatment/in treatment) as independent variables. Independent variables were selected based on the significant differences between groups identified through crosstabulations, viewed in light of theoretical and empirical relevance (the role of psychosocial and treatment characteristics in studies of treatment satisfaction) (Marchand et al., 2022; Trujols et al., 2017; Vanderplasschen et al., 2015). Only participants who remained in treatment at follow-up and had provided responses for treatment satisfaction were included in the final sample (*n* = 146) used in the logistic regression models. First, the odds ratios were calculated for each independent variable with the dependent variable, then all were combined in an adjusted model. The significance level was set to 0.05, with 95% confidence intervals. All analyses were conducted using SPSS, version 29 (IMB Corp, 2024).

## Results

### Treatment satisfaction

The sample (Table 1) consisted of 175 participants (48 women, 27.4%, 127 men, 72.6%). Of these, 161 (92.0%) were currently in OAT at the time of the study, one year following inclusion. The majority of participants reported that they were satisfied with treatment (54.9%). A substantial minority of participants experienced mixed treatment satisfaction (25.7%) and a slightly smaller group were dissatisfied with treatment (19.4%). There were no significant differences between age groups, nor men and women, in terms of distribution of satisfaction scores. However, the distribution differed depending on occupational status and housing situation, with treatment satisfaction being more common among those who were employed and/or studying, and those who had a stable housing situation.

**Table 1.** Demographic characteristics, substance use and well-being, and treatment characteristics across satisfaction groups at follow-up (n = 175).

		High satisfaction (*n* = 96, 54.9%)	Mixed (*n* = 45, 25.7%)	Low satisfaction (*n* = 34, 19.4%)	
		*n* (%)	*n* (%)	*n* (%)	*p*
Demographic characteristics					
	Age (at baseline)^a^ (*n* = 175)	39.1	36.8	40.5	0.213
	Gender (*n* = 175)				0.553
	Men	67 (52.8)	33 (26.0)	27 (21.3)	
	Women	29 (60.4)	12 (25.0)	7 (14.6)	
	Occupational status (*n* = 175)				**0**.**024**
	Employed	27 (75.0)	5 (13.9)	4 (11.1)	
	Not employed	69 (49.6)	40 (28.8)	30 (21.6)	
	Housing situation (*n* = 174)				**0**.**010**
	Stable	93 (57.8)	41 (25.5)	27 (16.8)	
	Unstable	3 (23.1)	3 (23.1)	7 (53.8)	
Substance use					
	Ongoing substance use (*n* = 160)				**0**.**004**
	Yes	65 (50.4)	33 (25.6)	31 (24.0)	
	No	24 (77.4)	7 (17.5)	0 (0)	
	Number of substances^a^ (*n* = 173)	2.1	2.6	3.7	**0**.**001**
	Intravenous substance use (*n* = 175)				**0**.**002**
	Yes	34 (44.7)	18 (23.7)	24 (31.6)	
	No	62 (62.6)	27 (27.3)	10 (10.1)	
	Substance use in social network (*n* = 175)				**0**.**002**
	Yes	17 (40.5)	9 (21.4)	16 (38.1)	
	No	79 (59.4)	36 (27.1)	18 (13.5)	
Well-being	Quality of relationship with self (*n* = 173)				0.334
	Good	56 (60.2)	22 (23.7)	15 (16.1)	
	Poor/mixed	40 (50.0)	21 (26.3)	19 (23.8)	
	Quality of friendships (*n* = 167)				**0**.**022**
	Good	69 (61.6)	26 (23.2)	17 (15.2)	
	Poor/mixed	22 (40.0)	17 (30.9)	16 (29.1)	
	Quality of contact with family (*n* = 170)				**0**.**006**
	Good	66 (62.9)	27 (25.7)	12 (11.4)	
	Poor/mixed	29 (44.6)	16 (24.6)	20 (30.8)	
	Anxiety symptoms (*n* = 175)				0.085
	Yes	31 (44.9)	23 (33.3)	15 (21.7)	
	No	65 (61.3)	22 (20.8)	19 (17.9)	
	Depressive symptoms (*n* = 175)				0.063
	Yes	22 (43.1)	19 (37.3)	10 (19.6)	
	No	74 (69.7)	26 (21.0)	24 (19.4)	
Treatment characteristics					
	OAT medication (*n* = 154)				0.622
	Mono buprenorphine	34 (56.7)	19 (31.7)	7 (11.7)	
	Buprenorphine/naloxone	34 (61.8)	12 (21.8)	9 (16.4)	
	Methadone	22 (56.4)	9 (23.1)	8 (20.5)	
	Supervised dispensing (*n* = 175)				0.436
	0-4 times/week	53 (59.6)	21 (23.6)	15 (16.9)	
	5-7 times/week	43 (50.0)	24 (27.9)	19 (22.1)	
	Weekly drug screenings (*n* = 120)				0.195
	Yes	47 (61.8)	19 (25.0)	10 (13.2)	
	No	21 (47.7)	12 (27.3)	11 (25.0)	
	Mental health treatment (*n* = 174)				0.083
	Yes	49 (62.0)	20 (25.3)	10 (12.7)	
	No	46 (48.4)	25 (26.3)	24 (25.3)	
	Individual treatment plan (*n* = 105)				**0**.**033**
	Yes	57 (65.5)	20 (23.0)	10 (11.5)	
	No	6 (33.3)	7 (38.9)	5 (27.8)	
	Patient care team (*n* = 134)				0.205
	Yes	72 (58.5)	31 (25.2)	20 (16.3)	
	No	4 (36.4)	3 (27.3)	4 (36.4)	
	Treatment interruptions (*n* = 165)				**0**.**002**
	Yes	12 (35.3)	8 (23.5)	14 (41.2)	
	No	77 (58.8)	35 (26.7)	19 (14.5)	
	Currently in treatment (*n* = 175)				**<0**.**001**
	Yes	95 (59.0)	42 (26.1)	24 (14.9)	
	No	1 (7.1)	3 (21.4)	10 (71.4)	

The significant *p* values are highlighted in bold text.

a Continuous variable.

Regarding substance use, there were no significant differences in the distribution of satisfaction scores between those who had ongoing substance use and those who did not currently use. However, treatment satisfaction was more common among those who used fewer substances, did not use substances intravenously, and were not involved in a substance-using social network. As for well-being, those who perceived their contact with family and the quality of their friendships more positively were more often satisfied with treatment. However, there were no significant differences in distribution of satisfaction scores between those who had experienced anxiety or depressive symptoms within the past four weeks compared to those who had not.

For treatment characteristics, there were no significant differences in satisfaction scores depending on type of OAT medication, supervised dispensing, weekly drug screenings, received mental health treatment or patient care teams. Participants who had an individual treatment plan were more often satisfied than those without an individual treatment plan. Treatment satisfaction was less common among those who had experienced treatment interruptions during the past year compared to those who had remained in treatment without interruptions.

### The role of psychosocial characteristics and treatment dimensions in treatment satisfaction

In the logistic regression analyses (Table 2), participants were grouped into two subsamples; those with mixed or low satisfaction (*n* = 79; 45.1%) and those satisfied with treatment (*n* = 96; 54.9%). In the binary logistic regression models, having an active occupational status, stable housing, good contact with family and friends, an individual treatment plan, and remaining in OAT at the time of follow-up were associated with increased odds for reporting treatment satisfaction, whereas ongoing substance use, being involved in a substance-using social network, and having experienced treatment interruptions were associated with decreased odds for reporting treatment satisfaction. In the adjusted, multivariable logistic regression model, both having an active occupational status and an individual treatment plan remained independently associated with increased odds for reporting treatment satisfaction, while ongoing substance use remained independently associated with decreased odds.

**Table 2.** Unadjusted and adjusted logistic regression analyses accounting for treatment satisfaction at follow-up (n = 146).

	Unadjusted ORs (95% CI)	Adjusted ORs (95% CI)
Age	1.007 (0.976–1.039)	1.011 (0.969–1.055)
Gender (ref. male)	1.367 (0.696–2.685)	1.735 (0.695–4.330)
Occupational status (ref. unemployed)	**5.574** (1.832–16.960)*	**3.475** (1.068–11.312)*
Housing situation (ref. unstable housing)	**4.559** (1.209–17.195)*	2.348 (0.515–10.699)
Ongoing substance use (ref. no)	**0.296** (0.119–0.736)*	**0.301** (0.105–0.858)*
Substance use in social network (ref. no)	**0.465** (0.229–0.942)*	0.843 (0.351–2.028)
Quality of contact with family (ref. poor/mixed)	**2.101** (1.120–3.941)*	1.915 (0.888–4.130)
Individual treatment plan (ref. no)	**2.387** (1.297–4.394)*	**2.371** (1.095–5.137)*
Treatment interruptions (ref. none)	**0.383** (0.175–0.838)*	0.696 (0.270–1.796)

The significant *p* values are highlighted in bold text.

*p values significant at the 0.05 level.

CI = confidence interval; OR = odds ratio.

## Discussion

In the present study, treatment satisfaction among patients in OAT was examined approximately one year after enrollment in a new treatment episode. More than half of the participants had high treatment satisfaction, one in four had mixed and one in five had low treatment satisfaction. This distribution of scores mirrors the distribution found in the Norwegian national annual survey in OAT in terms of high and mixed satisfaction (Nesse et al., 2024). However, the proportion of participants with low satisfaction was substantially higher in the present study and approximately equal in size to the proportion of missing responses in the national annual survey. While there were both satisfied and dissatisfied participants in this sample, it has been suggested that levels of satisfaction with OAT may depend on the methodological approach applied, with quantitative and global satisfaction measures more often yielding positive results (Trujols et al., 2014). Multidimensional measures and qualitative studies of treatment satisfaction may be valuable in pointing out potential strengths and obstacles in OAT, whether pertaining to the medical treatment or the various psychosocial interventions available (Trujols et al., 2014). Overall, previous studies conducted in other OAT contexts have indicated that most patients in OAT are generally fairly satisfied with the treatment that they receive, while at the same time as also highlighting treatment dimensions that are perceived as challenging (Marchand et al., 2022; Vanderplasschen et al., 2015).

In the present study, differences in treatment satisfaction were found depending on patients’ occupational status, housing situation, substance use, well-being, and treatment characteristics such as individual treatment plans and retention in treatment. In the adjusted logistic regression analyses, being employed and/or studying and having an individual treatment plan increased the odds that participants would be satisfied with OAT. Conversely, currently using substances decreased the odds for satisfaction with OAT. This suggests that treatment satisfaction varied by patients’ psychosocial situation while in treatment, as well as whether they received systematic, coordinated support. These results are in accordance with previous studies, which have implied that social functioning and involvement in treatment-related decision-making may play a positive role in treatment satisfaction (Trujols et al., 2012). Relatedly, engagement in OAT may often be followed by reduced substance use (Dong et al., 2020) and reduced substance use has been referred to as one of several relevant factors in perceptions of successful treatment (Reed et al., 2023). However, continued use while in OAT could indicate treatment needs, whether medical or psychosocial, that have been insufficiently addressed in treatment. In one study on treatment perceptions, the provision of housing support, support in engaging in meaningful activities, social networks and employment, and psychotherapy were sought-after treatment dimensions in OAT (Vanderplasschen et al., 2015).

Through the provision of systematic, coordinated support, important needs can be addressed, including those pertaining to establishing and maintaining a stable and satisfactory everyday life. This, in turn, may potentially contribute to positive treatment experiences for patients in OAT, with corresponding implications for engagement in treatment and treatment outcomes (Davis et al., 2020; Marchand et al., 2011; Miglietta et al., 2018). Given that substance use services generally, and OAT services specifically, increasingly seek to address patient perspectives and user involvement, it is vital to identify relevant needs and goals for treatment.

### Methodological strengths and limitations

The characteristics of the study sample appear to match those of the OAT population in Norway, as does the distribution of treatment satisfaction scores. This indicates that, despite the relatively small sample and subgroups, the results may be relevant within the Norwegian OAT context, without necessarily being generalizable to other OAT systems and services. Relatedly, the age of the dataset is a limitation given that OAT is constantly evolving. The study adds to the knowledge on factors that may promote or reduce treatment satisfaction. However, the use of a single, global measure of treatment satisfaction represents an important limitation of the study. In addition, several of the treatment dimensions that were used as independent variables were assessed with single items and simplistic formulations. Despite the risk of social desirability bias involved in the data being collected through interviews rather than through direct self-report, the fact that treatment satisfaction at follow-up was assessed by external researchers rather than treatment providers (as was the case for the baseline interviews) represents a potential strength. However, the use of compensation for participation in the study may have had implications for the choice to contribute to the study. Finally, the wide confidence intervals for occupational status and housing situation indicate a higher level of uncertainty for these variables in particular.

### Conclusions and implications for practice and further research

This study assessed treatment satisfaction among OAT patients 1 year after enrollment in a new treatment episode. Overall, the reported treatment satisfaction levels were high among most of the participants. Situational factors, as well as measures of coordinated, integrated support, were found to play a role in treatment satisfaction. Coordinated, integrated support, such as through the application of individual treatment plans and patient care teams, may be useful in explicitly addressing the patients’ situation and everyday life with aims of promoting well-being.

There is a need for multidimensional and contextually anchored measures of treatment satisfaction in OAT to better capture the complexity of treatment experiences, and to be able to identify potential facilitators for and barriers to treatment satisfaction. In future research, additional measures of coordinated, integrated support should be included. Future studies could extended long-term follow-ups to be able to follow changes and consistencies in treatment satisfaction trajectories in OAT. Treatment satisfaction in OAT is relevant both in research and in clinical practice, in the latter case as a construct for providers to initiate conversations regarding treatment needs and treatment goals, and to facilitate user involvement.

## References

[bibr1-14550725251351711] Apantaku-Olajide T. Ducray K. Byrne P. Smyth B. P. (2012). Perception of unmet needs and association with benzodiazepine misuse among patients on a methadone maintenance treatment programme. The Psychiatrist, 36(5), 169–174. 10.1192/pb.bp.111.036616

[bibr2-14550725251351711] Davis E. L. Kelly P. J. Deane F. P. Baker A. L. Buckingham M. Degan T. Adams S. (2020). The relationship between patient-centered care and outcomes in specialist drug and alcohol treatment: A systematic literature review. Substance Abuse, 41(2), 216–231. 10.1080/08897077.2019.1671940 31638870

[bibr3-14550725251351711] De Maeyer J. Vanderplasschen W. Broekaert E. (2009). Exploratory study on drug users’ perspectives on quality of life: More than health-related quality of life? Social Indicators Research, 90, 107–126. 10.1007/s11205-008-9315-7

[bibr4-14550725251351711] Dong H. Hayashi K. Milloy M. J. DeBeck K. Singer J. Wong H. Wood E. Kerr T. (2020). Changes in substance use in relation to opioid agonist therapy among people who use drugs in a Canadian setting. Drug and Alcohol Dependence, 212. 10.1016/j.drugalcdep.2020.108005 PMC7462099 32370932

[bibr5-14550725251351711] Ellefsen R. Wüsthoff L. E. C. Arnevik E. A. (2023). Patients’ satisfaction with heroin-assisted treatment: A qualitative study. Harm Reduction Journal, 20(1), 73. 10.1186/s12954-023-00808-8 37312181 PMC10265766

[bibr6-14550725251351711] European Monitoring Centre for Drugs and Drug Addiction. (2023). Viral hepatitis elimination barometer among people who inject drugs in Europe. https://www.emcdda.europa.eu/publications/data-factsheet/viral-hepatitis-elimination-barometer-among-people-who-inject-drugs-in-europe_en

[bibr7-14550725251351711] Friedrichs A. Spies M. Härter M. Buchholz A. (2016). Patient preferences and shared decision making in the treatment of substance use disorders: A systematic review of the literature. PloS One, 11(1), 1–18. 10.1371/journal.pone.0145817 PMC4701396 26731679

[bibr8-14550725251351711] Hall N. Le L. Majmudar I. Mihalopoulos C. (2021). Barriers to accessing opioid substitution treatment for opioid use disorder: A systematic review from the client perspective. Drug and Alcohol Dependence, 211(4), 108651. doi: 10.1016/j.drugalcdep.2021.108651 33667783

[bibr9-14550725251351711] Hansson L. Björkman T. Priebe S. (2007). Are important patient-rated outcomes in community mental health care explained by only one factor? Acta Psychiatrica Scandinavia, 116(2), 113–118. 10.1111/j.1600-0447.2007.01005.x 17650272

[bibr10-14550725251351711] Hooker S. A. Sherman M. D. Lonergan-Cullum M. Nissly T. Levy R. (2022). What is success in treatment for opioid use disorder? Perspectives of physicians and patients in primary care settings. Journal of Substance Abuse Treatment, 141, 1–7. 10.1016/j.jsat.2022.108804 35643586

[bibr11-14550725251351711] IBM Corp. (2024). IBM SPSS Statistics for Windows (Version 29.0) [Computer software]. IBM Corp.

[bibr13-14550725251351711] Marchand K. Oviedo-Joekes E. Guh D. Brissette S. Marsh D. C. Schechter M. T. (2011). Client satisfaction among participants in a randomized trial comparing oral methadone and injectable diacetylmorphine for long-term opioid-dependency. BMC Health Services Research, 11, 1–10. 10.1186/1472-6963-11-174 21791093 PMC3161847

[bibr14-14550725251351711] Marchand K. Palis H. Guh D. Lock K. MacDonald S. Brissette S. Marsh D. C. Harrison S. Schechter M. T. Oviedo-Joekes E. (2022). A multi-methods and longitudinal study of patients’ perceptions in injectable opioid agonist treatment: Implications for advancing patient-centered methodologies in substance use research. Journal of Substance Abuse Treatment, 132, 1–10. 10.1016/j.jsat.2021.108512 34098207

[bibr15-14550725251351711] Marchand K. Palis H. Peng D. Fikowski J. Harrison S. Spittal P. Schechter M. T. Oviedo-Joekes E. (2015). The role of gender in factors associated with addiction treatment satisfaction among long-term opioid users. Journal of Addiction Meidicine, 9(5), 391–398. 10.1097/ADM.0000000000000145 PMC4605272 26335006

[bibr16-14550725251351711] Medved D. Clausen T. Bukten A. Bjørnestad R. Muller A. E. (2020). Large and non-specific somatic disease burdens among ageing, long-term opioid maintenance treatment patients. Substance Abuse: Treatment, Prevention, and Policy, 15, 1–9. 10.1186/s13011-020-00311-4 33198799 PMC7667746

[bibr17-14550725251351711] Migchels C. Zerrouk A. Crunelle C. L. Matthys F. Gremeaux L. Fernandez K. Antoine J. van den Brink W. Vanderplasschen W. (2023). Patient reported outcome and experience measures (PROMs and PREMs) in substance use disorder treatment services: A scoping review. Drug and Alcohol Dependence, 111017. 10.1016/j.drugalcdep.2023.111017 37995391

[bibr18-14550725251351711] Miglietta E. Belessiotis-Richards C. Ruggeri M. Priebe S. (2018). Scales for assessing patient satisfaction with mental health care: A systematic review. Journal of Psychiatric Research, 100, 33–46. 10.1016/j.jpsychires.2018.02.014 29482063

[bibr19-14550725251351711] Muller A. E. Bjørnestad R. Clausen T. (2018). Dissatisfaction with opioid maintenance treatment partly explains reported side effects of medications. Drug and Alcohol Dependence, 87, 22–28. 10.1016/j.drugalcdep.2018.02.018 29626742

[bibr20-14550725251351711] Muller A. E. Skurtveit S. Clausen T. (2016). Validating the generic quality of life tool “QOL10” in a substance use disorder treatment cohort exposes a unique social construct. BMC Medical Research Methods, 16, 1–8. 10.1186/s12874-016-0163-x PMC4878076 27216750

[bibr21-14550725251351711] Müller O. Baumann C. Patrizio P. D. Viennet S. Vlamynck G. Collet L. Clerc-Urmes I. Schwan R. Bourion-Bedes S. (2020). Patients’ early satisfaction with care: A predictor of health-related quality of life change among outpatients with substance dependence. Health and Quality of Life Outcomes, 18, 1–11. 10.1186/s12955-019-1245-3 31910879 PMC6947996

[bibr22-14550725251351711] Nesse L. Lobmaier P. P. Skeie I. Lillevold P. H. Clausen T. (2024). Statusrapport 2023: Tjuefem år med legemiddelassistert rehabilitering (LAR) [Status report 2023: Twenty-five years of opioid agonist treatment (OAT)]. [Internet]. Norwegian Centre for Addiction Research, University of Oslo. (Cited 2025 Jan 30). https://www.med.uio.no/klinmed/forskning/sentre/seraf/publikasjoner/rapporter/2024/seraf-rapport-nr-2-2024-statusrapport-2023.pdf

[bibr23-14550725251351711] Norwegian Directorate of Health. (2017). Veileder om oppfølging av personer med store og sammensatte behov [Guidelines for the rehabilitation of persons with complex needs]. [Internet]. (Cited 2024 March 26). https://www.helsedirektoratet.no/veiledere/oppfolging-av-personer-med-store-og-sammensatte-behov/strukturert-oppfolging-gjennom-tverrfaglige-team

[bibr24-14550725251351711] Norwegian Directorate of Health. (2018). Om individuell plan og koordinator – formål og rettigheter [On individual treatment plans and treatment coordinators – purpose and rights]. [Internet]. (Cited 2024 Mar 26). https://www.helsedirektoratet.no/veiledere/rehabilitering-habilitering-individuell-plan-og-koordinator/individuell-plan-og-koordinator/om-individuell-plan-og-koordinator-formal-og-rettigheter

[bibr25-14550725251351711] Reed M. K. Smith K. R. Ciocco F. Hass R. W. Cox A. L. Kelly E. L. Weinstein L. C. (2023). Sorting through life: Evaluating patient-important measures of success in a medication for opioid use disorder (MOUD) treatment program. Substance Abuse: Treatment, Prevention and Policy, 18(1), 1–12. 10.1186/s13011-023-00514-5 36641478 PMC9839958

[bibr26-14550725251351711] Trujols J. Garijo I. Sinol N. del Pozo J. Portella M. J. Cobos J. P. (2012). Patient satisfaction with methadone maintenance treatment: The relevance of participation in treatment and social functioning. Drug and Alcohol Dependendence, 123(1-13), 41–47. 10.1016/j.drugalcdep.2011.10.014 22071121

[bibr27-14550725251351711] Trujols J. Gonzalez-Saiz F. Manresa M. J. Alcaraz S. Batlle F. Duran-Sindreu S. de los Cobos J. P. (2017). Patient perception of methadone dose adequacy in methadone maintenance treatment: The role of perceived participation in dosage decisions. Patient Education and Counselling, 100(5), 981–986. 10.1016/j.pec.2016.12.001 27988071

[bibr28-14550725251351711] Trujols J. Iraurgi I. Oviedo-Joekes E. Guàrdia-Olmos J. (2014). A critical analysis of user satisfaction surveys in addiction services: Opioid maintenance treatment as a representative case study. Patient Preference and Adherence, 107–117. 10.2147/PPA.S52060 24482571 PMC3905099

[bibr29-14550725251351711] Vanderplasschen W. Naert J. Vander Laenen F. De Maeyer J. (2015). Treatment satisfaction and quality of support in outpatient substitution treatment: Opiate users’ experiences and perspectives. Drugs: Education, Prevention and Policy, 22(3), 272–280. 10.3109/09687637.2014.981508

[bibr30-14550725251351711] Windle E. Tee H. Sabitova A. Jovanovic N. Priebe S. Carr C. (2020). Association of patient treatment preference with dropout and clinical outcomes in adult psychosocial mental health interventions: A systematic review and meta-analysis. JAMA Psychiatry, 77(3), 294–302. 10.1001/jamapsychiatry.2019.3750 31799994 PMC6902231

